# Decoding canine parvovirus: biomarkers for diagnosis and advances in vaccine development to address emerging challenges

**DOI:** 10.3389/fvets.2025.1624275

**Published:** 2025-08-06

**Authors:** Hao Li, Shengnan Li, Ye Pan, Qiumei Shi

**Affiliations:** ^1^The Laboratory Animal Research Center, Jiangsu University, Zhenjiang, China; ^2^School of Medicine, Southeast University, Nanjing, China; ^3^Hebei Provincial Key Laboratory of Preventive Veterinary Medicine, College of Animal Science and Technology, Hebei Normal University of Science and Technology, Qinghuangdao, China

**Keywords:** canine parvovirus, biomarker, enteritis, myocarditis, vaccine

## Abstract

Canine parvovirus (CPV) enteritis is a highly contagious disease caused by CPV, primarily affecting canids and posing a severe threat to their health. Prevention of CPV infection relies mainly on attenuated live vaccines, subunit vaccines, and inactivated vaccines, all of which can induce protective immunity. However, the incomplete protective efficacy provided by some vaccines and fatalities in dogs due to immunization failure have significantly impacted the dog-breeding industry. Early diagnosis is crucial for timely treatment, but traditional detection methods like hemagglutination inhibition tests often lead to misdiagnosis, delaying therapy. In recent years, with in-depth research, novel diagnostic techniques and advanced vaccines have been continuously developed, achieving notable progress. Against this backdrop, this review summarizes the advancements in CPV vaccines based on domestic and international studies on CPV biomarkers and vaccination strategies. Specifically, the etiological characteristics of CPV exhibit dynamic evolutionary trends. Key amino acid mutations in the VP2 capsid protein (e.g., D426E) drive viral antigenic drift, giving rise to variants such as CPV-2a, 2b, and 2c. CPV-2c has become the predominant strain in Europe and South America, with conformational changes in its antigenic epitopes reducing neutralizing antibody titers induced by traditional vaccines by 4–8 folds. In terms of biomarkers, CPV infection triggers multi-systemic changes, including blood components (e.g., hematocrit, white blood cell count, platelet count), biochemical indicators (sodium/chloride electrolytes, hepatic/renal function markers), C-reactive protein (CRP), intestinal markers (I-FABP, TFF-3), and cardiac markers (cTnI, CK-MB). These markers are used to assess infection status, disease severity, and prognosis (e.g., CRP > 92.4 mg/L predicts mortality with 91% sensitivity). In vaccine development, attenuated live vaccines remain effective for preventing CPV enteritis but face challenges like maternal antibody interference and reduced efficacy caused by viral mutation. Inactivated vaccines offer high safety but low immunogenicity, requiring multiple vaccine administrations. DNA vaccines and subunit vaccines (e.g., virus-like particles self-assembled by VP2 protein) show promising prospects, with novel CPV-2c vaccines overcoming maternal antibody interference in puppies. However, the high mutation rate of CPV (0.0045 substitutions/site/year for VP2 gene) delays the updating of traditional vaccine strain updating, necessitating accelerated development of vaccines targeting prevalent strains (e.g., CPV-2c). Future research should focus on viral mutation monitoring, precision diagnostic technology, and strain-matched vaccine development to enhance CPV control efficiency.

## 1 Etiology

CPV is the etiological agent responsible for canine parvovirus disease, with its pathogenetic characteristics exhibiting a dynamic evolutionary trajectory. The virus was first isolated and identified in dogs with acute enteritis in 1978. Genomic analysis indicates that it originated from host-adaptive mutations in feline panleukopenia virus (FPLV). During the continuous evolution, key amino acid mutations in the VP2 capsid protein drive viral antigenic drift, giving rise to successive antigenic variants, including CPV-2a (1980), CPV-2b (1984), and CPV-2c (2000) (1–3), which has led to genetic lineage differentiation that spreads globally (4). The mutation of aspartic acid to glutamic acid at position 426 (D426E) in the VP2 protein is the hallmark change that distinguishes CPV-2c from the classic strains. This site is located in the antigenic epitope region of the viral capsid and directly affects the binding efficiency between the virus and the host transferrin receptor (TfR). This structural alteration not only enhances the virus’s affinity for canine intestinal epithelial cells but also induces conformational changes in its antigenic epitopes, leading to a 4- to 8-fold reduction in the neutralizing antibody titers induced by traditional vaccines (5). Epidemiological surveys have shown that CPV-2c has replaced the classic strains as the predominant strain in regions such as Europe and South America, whereas CPV-2a subtype the mainstream in Asia (6–8).

## 2 Biomarkers

**Figure 1:**
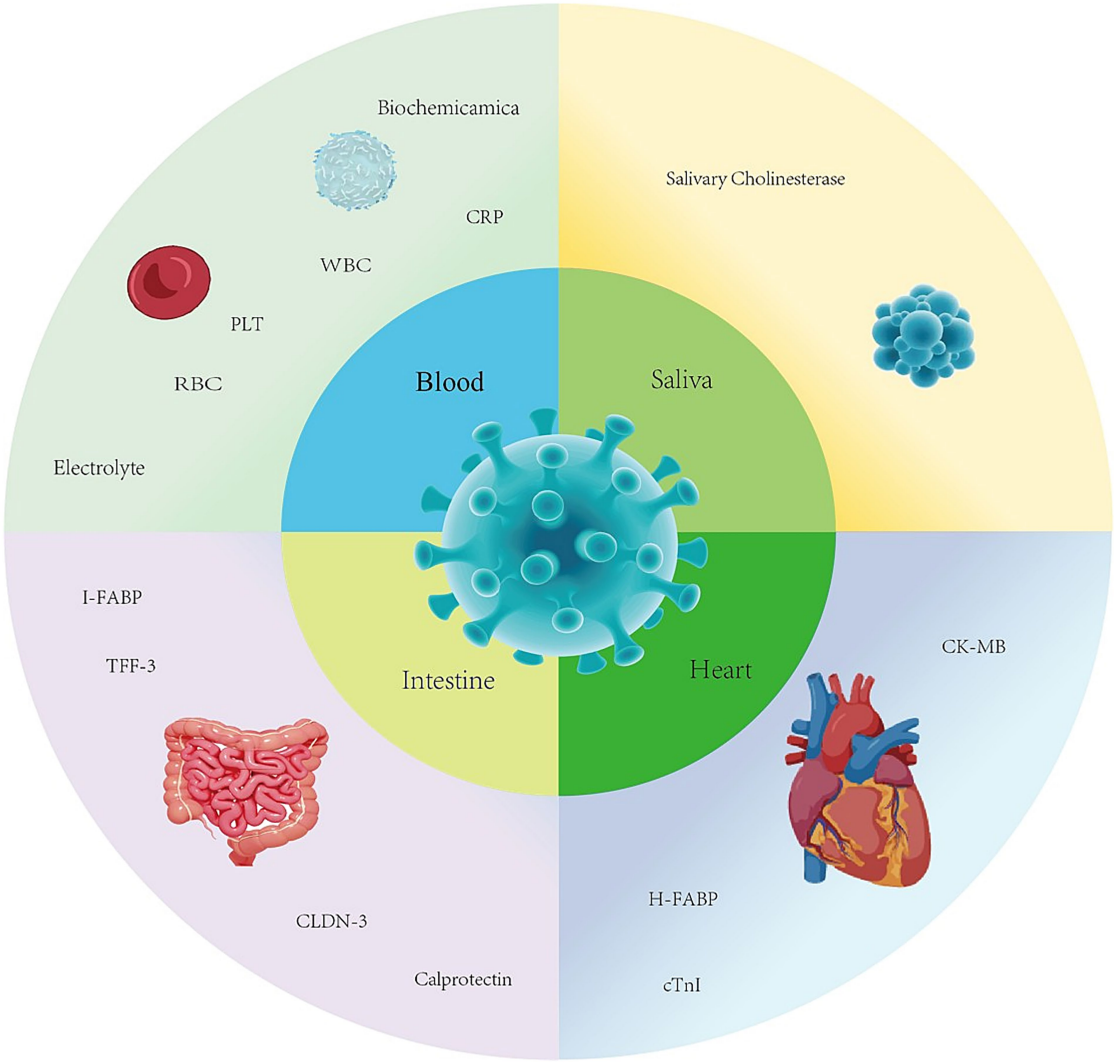
Biomarkers associated with canine parvovirus.

Biomarkers can serve as indicators of a biological state, reflecting physiological or pathological processes (9) and can be objectively measured and evaluated. In the context of CPV infection, these biomarkers enhance disease assessment by enabling more accurate judgments and targeted interventions, thereby helping to determine infection status, disease severity, and prognosis in affected dogs (10). Additionally, they facilitate decision-making for dog owners during discussions on treatment plans or euthanasia (11). The infection of Canine Parvovirus is associated with biomarker changes across multiple systems, as shown in the diagram (Figure 1).

### 2.1 Changes in blood components: a window into canine parvovirus enteritis

#### 2.1.1 Changes in erythrocyte-related parameters

CPV induces dynamic alterations in erythrocyte-related parameters, with these changes closely linked to disease progression and prognosis. In the early infection stage, affected dogs exhibit severe vomiting and diarrhea, leading to substantial fluid loss and subsequent hemoconcentration. This is manifested by significant elevations in hematocrit (HCT) and red blood cell count (RBC). The elevation of erythrocyte parameters at this stage represents a typical compensatory response to dehydration, with the degree of HCT increase serving as an important indicator for assessing the severity of dehydration and guiding in clinical emergency fluid resuscitation.

As the disease advances to the mid-to-late stages, persistent gastrointestinal bleeding and mucosal damage result in impaired erythropoiesis and enhanced erythrocyte destruction, ultimately leading to anemia (12). Laboratory findings demonstrate a decrease in hemoglobin (HGB) concentration, along with concurrent reductions in mean corpuscular volume (MCV), mean corpuscular hemoglobin (MCH), and mean corpuscular hemoglobin concentration (MCHC) (13). This synchronized decline in multiple parameters indicates non-regenerative anemia, reflecting the dual pathological mechanisms of impaired bone marrow hematopoietic function and chronic blood loss.

#### 2.1.2 Leukocyte count

CPV infection elicits profound leukocyte abnormalities. Within the initial 4–5 days of disease onset, the total leukocyte count (WBC) undergoes a dramatic decline due to viral-mediated damage on the gastrointestinal tract and immune system. This results in a substantial reduction in leukocyte production by the bone marrow, compounded by extensive leukocyte consumption during the inflammatory response to intestinal damage. The WBC can plummet to 500–2,000 cells/mm^3^. In differential counts, neutrophil (NEU) proportions decrease, while lymphocyte (LYM) proportions increases relatively. Abnormalities may also involve eosinophils and basophils, with elevated band cells and monocyte counts. For example, early in the disease, neutrophils may drop from approximately 60 to 30%, while lymphocytes rise from approximately 30 to 50% (14). This leukopenia critically impairs immune function. Neutrophils, lymphocytes, and monocytes collaborate to combat viral invasion: neutrophils phagocytose and kill the virus, lymphocytes mediate adaptive immune responses against infected cells, and monocytes contribute to antigen presentation. Babul R. Paul et al. reported that affected dogs had a median total leukocyte count (TLC) of 2.35 × 10^3^/μl, significantly lower than that of both the systemic inflammatory response syndrome negative group (4.1 × 10^3^/μl) and healthy controls (10.00 × 10^3^/μl) (14). This leukopenia stems from bone marrow suppression, lymphoid tissue depletion, and heightened demand for leukocyte at inflammatory sites.

Leukopenia is also correlated with insufficient endothelial activation-related cytokines and proteases. A decline in leukocyte numbers impairs their capacity to secrete cytokines and proteases that stimulate endothelial activation. Under physiological conditions, these factors promote endothelial cell activation, enhance vascular endothelial defense, and facilitate leukocyte adhesion/migration to infection foci. Insufficient secretion disrupts endothelial activation, thereby compromising the entire immune response cascade. Circulating endothelial adhesion molecules concentrations exhibit complex dynamics (15). Studies show that during CPV enteritis onset in dogs, serum levels of intercellular adhesion molecule-1 (ICAM-1) and vascular cell adhesion molecule-1 (VCAM-1)—key endothelial adhesion molecules—do not increase; they may even decrease. Additionally, high-mobility group box protein 1 (HMGB-1) remains unchanged. This suggests an altered endothelial activation status (16, 17). Under typical infection or inflammatory responses, endothelial cell activation upregulates adhesion molecule expression, promoting leukocyte recruitment to inflamed sites. In contrast, CPV infection elicits the opposite response, likely due to complex immune regulatory mechanisms triggered by viral infection that impair the regulation of endothelial cell responses (18).

These adhesion molecule concentration changes potentially impact leukocyte adhesion and migration. These are critical steps in the immune response that enable leukocytes to reach infection sites and exert effector functions. Reduced endothelial adhesion molecule levels weaken leukocyte-endothelial cell interactions, compromising the efficiency of migration to infected tissues (e.g., intestinal mucosa). This may sustain viral replication in the intestine and hinder inflammatory response control. For example, studies have shown that pharmacological interference with endothelial adhesion molecule expression alters leukocyte migratory capacity, influencing disease progression (19). Thus, investigating the dynamics, tissue expression, and cytokines associated with endothelial adhesion molecules in CPV infection is of great significance. Such research could deepen our understanding of CPV pathogenesis and facilitate the development of novel therapeutic strategies. For instance, identifying agents that regulate endothelial adhesion molecule expression to promote effective leukocyte adhesion/migration may enhance the host immune response to CPV, improving clinical outcomes in affected dogs.

#### 2.1.3 Platelet count

CPV infection induces significant platelet abnormalities, with infected dogs typically exhibiting marked thrombocytopenia. This phenomenon stems from two primary mechanisms: first, direct viral-mediated destruction of platelets, leading to reduced platelet counts; and second, the formation of immune complexes following viral infection, which may indirectly damage platelets and further exacerbate thrombocytopenia (14). As a critical component of the coagulation cascade, platelet depletion significantly elevates the bleeding risk in affected dogs, particularly when accompanied by severe diarrhea and vomiting. In this scenario, gastrointestinal mucosal inflammation and damage render the mucosa fragile, substantially increasing the propensity for hemorrhage. Such bleeding not only aggravates anemia and debilitation in dogs but also predisposes them to secondary infections, complicating the clinical course and potentially threatening survival. Epidemiological data show that 30–40% of CPV-infected dogs develop varying degrees of thrombocytopenia (14, 20). Platelet count reduction correlates positively with bleeding risk—i.e., lower platelet counts are associated with higher bleeding risk. Additionally, thrombocytopenia may impede the overall recovery of affected dogs: bleeding and anemia compromise immune function and tissue repair, diminishing the ability to combat viral and other pathogenic invasions. Thus, monitoring platelet counts and implementing prompt interventions are crucial in the management of CPV infection in dogs.

#### 2.1.4 Fluctuations in biochemical indicators

Following CPV infection, viral variant dynamics and host immune status have led to increased attention to the clinical manifestations and biochemical indicators of infected dogs. Studies have shown that approximately 40% of infected dogs exhibit elevated serum sodium (Na^+^) and chloride (Cl^−^) concentrations, with calcium (Ca^2+^) levels also potentially mildly elevated. These changes are closely associated with dehydration and electrolyte disturbances. Research has demonstrated a significant positive correlation between serum sodium concentration and dehydration severity in CPV-infected dogs (*p* < 0.01), and dogs with hypernatremia have a mortality rate 1.8-fold higher than that of normal controls (21). Another study suggests that virus-mediated destruction of intestinal crypt epithelia activates the sodium-hydrogen exchanger (NHE3), exacerbating intestinal sodium absorption dysfunction. Although CPV primarily targets intestinal and myocardial cells, the systemic inflammatory response it elicits can involve the liver and kidneys, leading to abnormalities in hepatic and renal function biomarkers. For example, elevations in alanine aminotransferase (ALT), aspartate aminotransferase (AST), and creatinine (CREA) indicate hepatic and renal damage, warranting close monitoring of liver and kidney function during treatment (22). In one cohort of affected dogs, approximately 40% had serum sodium levels above the normal range, with ALT levels increasing by an average of 2-3-fold. Franzo et al. confirmed via histopathological analysis that CPV-2c variant viral DNA was present in the livers of infected dogs, with viral load positively correlated with ALT elevation (21). Additionally, the decline in glomerular filtration rate (GFR) in infected dogs is directly related to hypovolemia and inflammatory mediators (such as TNF-α). The average fluid loss in CPV-infected dogs can reach 8–10% of body weight, necessitating intravenous fluid resuscitation to correct hypovolemia (23).

#### 2.1.5 C-reactive protein (CRP)

As an acute-phase reactant, CRP shows a rapid increase following CPV infection (24). Parvovirus infection triggers an intense intestinal inflammatory response, stimulating hepatocytes to synthesize CRP and leading to marked serum CRP elevation. CRP binds to calcium-dependent complexes that activate the complement system, release inflammatory mediators, and promote leukocyte adhesion/phagocytosis, thereby lysing target cells and playing a pivotal role in the inflammatory cascade (25). Moreover, serum CRP concentration is key to predicting the prognosis of CPV enteritis (15). Canine serum CRP normally remains extremely low (<10 mg/L), but CPV infection induces a dramatic surge. Engelbrecht et al. reported that CPV-infected dogs had significantly higher serum CRP than healthy controls (statistical significance: *p* < 0.001, median values unspecified), confirming CRP as a sensitive inflammatory marker for CPV infection (26, 27). CRP levels correlate closely with survival probability, serving as an important prognostic indicator. Further studies show that CRP outperforms ceruloplasmin, haptoglobin, and albumin in distinguishing between surviving and non-surviving dogs (28). A CRP threshold >92.4 mg/L yields 91% sensitivity for predicting mortality, providing valuable prognostic information despite suboptimal specificity (29).du Preez et al. found CRP negatively correlated with segmented neutrophil counts (r_s_ = −0.472) and positively correlated with band neutrophil counts (r_s_ = 0.678), suggesting that elevated CRP reflects neutrophil consumption and bone marrow release of immature cells (30). In clinical practice, veterinarians can adjust treatment plans in a timely manner by monitoring the changes in serum CRP concentration in affected dogs. If the CRP concentration continues to rise, it may indicate a poor prognosis, necessitating intensified treatment or the exploration of new therapeutic approaches; if the gradually decreases, it indicates that the treatment measures are effective and the prognosis for the affected dog is relatively better (31).

### 2.2 Intestinal biomarkers

Intestinal biomarkers play a pivotal role in reflecting disease status in CPV enteritis. In research on canine diseases, intestinal fatty acid-binding protein (I-FABP), trefoil factor 3 (TFF-3), and claudin-3 (CLDN-3) are closely associated to intestinal health (32, 33). Serum I-FABP is significantly elevated at baseline (0 h) in affected dogs, as intestinal mucosal damage triggers the release of I-FABP from intestinal epithelial cells into the bloodstream (34, 35). In CPV infection models, serum I-FABP levels in affected dogs increase several-fold compared to healthy controls at baseline, establishing I-FABP as a reliable indicator of intestinal injury with mortality predictive value (34). TFF-3 shows a significant elevation at both baseline and 48 h in affected dogs. Secreted by intestinal goblet cells, TFF-3 is critical for intestinal mucosal repair, participating in the repair process during CPV-induced intestinal damage and exhibiting value in predicting mortality at 48 h. CLDN-3 levels remain unchanged in affected dogs, likely due to early-stage disease where intestinal barrier function is not severely compromised. As the disease progresses, CLDN-3 may serve as a potential marker for monitoring intestinal barrier dysfunction if barrier integrity is disrupted (36).

Fecal calprotectin is a heterodimer of calcium- and zinc-binding proteins S100A8 and S100A9. Calgranulin C, like calprotectin, belongs to the S100/calgranulin subfamily of Ca2^+^-binding proteins and plays an important role in innate immune responses. Calgranulin C and calprotectin are released by activated macrophages and/or neutrophils, accumulate at sites of inflammation, and are subsequently excreted in feces through the mucosa. Due to the resistance of S100 proteins to fecal microbial degradation, they can exist stably in feces, making them suitable markers for intestinal inflammation. Heilmann et al. demonstrated that S100A12, S100A8 and S100A9, and macrophages are involved in the pathogenesis of canine inflammatory bowel disease, but further investigation is warranted (37).

### 2.3 Cardiac biomarkers

Cardiac biomarkers also provide important clues for assessing disease severity in CPV infections (38, 39). Creatine kinase MB (CK-MB) is significantly elevated at baseline (0 h) in affected dogs, as viral infection may lead to myocardial cell damage, prompting the release of CK-MB from myocardial cells into the bloodstream (40, 41). No significant differences are observed at 48 h, indicating that the myocardial cell damage may have been alleviated to some extent or ceased to deteriorate over time. Kocatürk et al. found that CK-MB levels in CPV-infected dogs upon admission were significantly higher than those in healthy dogs, suggesting that the virus may directly or indirectly disrupt myocardial cell membrane permeability, leading to the release of intracellular enzymes into the blood (42).

Cardiac troponin I (cTnI), as a specific biomarker for myocardial damage, is rapidly released into the blood following myocardial injury, with its dynamic changes can accurately reflecting the severity and progression of myocardial damage. Studies have shown that in myocarditis caused by CPV infection, cTnI is significantly elevated at both the early stage of infection (0 h) and 48 h, providing evidence of viral effects on myocardial cells, which is highly correlated with the pathological process of acute myocarditis (34). Persistent elevation of cTnI not only indicates progressive worsening of myocardial damage but may also suggest irreversible myocardial necrosis or secondary lesions, consistent with the cTnI release patterns observed in human and animal myocarditis models (43). It is worth noting that the dynamic changes of cTnI in CPV myocarditis may be influenced by the viral replication phase and the host immune response. For example, puppies, with rapidly dividing myocardial cells, are more susceptible to CPV attack, leading to cTnI release prior to clinical symptom onset, whereas the presence of maternal antibodies may delay or mask detection results. These findings echo research on human viral myocarditis, indicating the conservation of cTnI release mechanisms across species (44). Future studies may combine multi-omics technologies to further elucidate the molecular regulatory pathways of cTnI in virus-host interactions, thereby optimizing clinical diagnosis and treatment strategies.

Heart-type fatty acid-binding protein (H-FABP) is rapidly released into the bloodstream in the early stages of myocardial damage. However, due to its small molecular weight, it is quickly metabolized and cleared by the kidneys. It can be released into the blood within 1–3 h after myocardial cell injury, reaching a peak at 2–5 h, however, with a half-life of only 0.3 h, it returns to normal levels within 24 h (34). This characteristic makes it a sensitive indicator of early myocardial damage but may also result in a short detection window due to rapid metabolism.

Hematological indicators play a crucial role in the diagnosis and treatment of CPV enteritis. By closely monitoring indicators such as red blood cells, white blood cells, and platelets, as well as assessing CRP, intestinal biomarkers, and cardiac biomarkers, veterinarians can accurately assess the severity of the disease, the status of the inflammatory response, and the prognosis in affected dogs. These indicators serve as key guiding, providing veterinarians with extremely important evidence for formulating scientific and rational treatment plans.

### 2.4 Changes in salivary cholinesterase

The mechanisms underlying the changes in salivary cholinesterase induced by CPV infection are rather complex and involve multiple aspects. Following viral invasion, neurotransmitter metabolism in dogs may be disrupted, thereby interfering with cholinesterase synthesis, transport, and metabolic pathways (45). Inflammatory responses, a significant pathophysiological process in CPV infection, may lead to elevated levels of cholinesterase in saliva due to the release of inflammatory mediators, which alters cholinesterase synthesis and transport pathways (46). The immune response triggered by viral infection may activate relevant signaling pathways, prompting organs such as the liver to synthesize more cholinesterase and release it into the bloodstream, thereby influencing salivary cholinesterase levels. Direct or indirect damage to the nervous system by the virus may also cause neurotransmitter imbalances, thereby causing changes in salivary cholinesterase. From a pathophysiological perspective, changes in salivary cholinesterase are closely related to disease severity. As the condition worsens, metabolic disturbances and inflammatory reactions intensify, which may further disrupt the metabolic balance of cholinesterase and cause a sustained increase in salivary cholinesterase levels (47). Kocatürk et al. reported that salivary cholinesterase (Ch) showed a moderate positive correlation with serum CRP, a weak positive correlation with clinical scores, and a weak negative correlation with white blood cell counts; serum Ch, by contrast, showed only weak positive correlations with clinical scores and CRP, and a weak negative correlation with white blood cell counts (42). In addition, clinical scores and white blood cell counts have significant predictive value for mortality, while the predictive value of other indicators is insignificant. However, due to the small number of deceased animals, these results need to be interpreted with caution to avoid biases caused by insufficient sample size. This study provides new insights and perspectives for the diagnosis and treatment of CPV infection, with salivary Ch potentially serving as a biomarker for disease severity in CPV-infected dogs. However, the study also has certain limitations, such as a small sample size, which may not fully represent the entire canine population. Further studies need to expand the sample size and conduct more in-depth and comprehensive validation to enhance the reliability and generalizability of the research findings, thereby providing stronger support for the clinical diagnosis and treatment of CPV infection.

### 2.5 The biomarkers for prognostic evaluation of CPV infection

CPV is a common and highly lethal disease in dogs, with prognostic assessment being crucial for formulating clinical treatment strategies. The dynamic monitoring of biomarkers provides an objective basis for precise evaluation. Key hematological and inflammatory biomarkers associated with CPV infection, including their critical thresholds and clinical implications, are summarized in Table 1. The total WBC can drop sharply to 0.5–2.0 × 10^3^/μl within 48 h after infection. The mortality rate of dogs with WBC < 1.0 × 10^3^/μl is 4.7 times higher than that of those with WBC > 5.0 × 10^3^/μl. The proportion of immature neutrophils (bands cells) > 15% increases the risk of multiple organ failure by 3 times. The failure of WBC to rise after 48 h of treatment indicates a poor prognosis (6, 48). Platelet count < 50 × 10^3^/μl is an independent risk factor for a poor prognosis. When platelet count is < 20 × 10^3^/μl, the gastrointestinal mucosal bleeding rate reaches 68%, and the survival rate is less than 20%. The daily decline > 30% in platelet count can better predict the bleeding risk than the absolute value (49). CRP rises to 100–300 mg/L within 24 h after infection. CRP > 92.4 mg/L can predict 91% of deaths. When CRP continues to be >150 mg/L, the survival rate is less than 15%. The decline < 30% in CRP after 48 h of treatment indicates that the inflammation remains uncontrolled. When CRP > 92.4 mg/L is combined with WBC < 1.0 × 10^3^/μl, the mortality rate is as high as 82% (25, 50). I-FABP > 3 ng/ml indicates damage to the intestinal mucosal barrier, and > 10 ng/ml is associated with a mortality rate of 75%, which is positively correlated with the need for fluid resuscitation. TFF-3 > 5 ng/ml indicates the activation of intestinal repair. If it does not increase within 48 h, the mortality rate increases by 3 times. The combination of the two can evaluate the dynamic balance of intestinal injury and repair (16). cTnI > 0.1 ng/ml (adult dogs) or > 0.2 ng/ml (puppies) indicates myocardial necrosis. When cTnI continues to be > 0.5 ng/ml, the mortality rate within 24 h reaches 60%. CK-MB > 200 U/L indicates myocardial injury, and the failure to decrease within 48 h indicates poor prognosis. The combination of these two biomarkers can make the diagnostic rate of myocarditis reach 92% (51, 52). In addition, the increase in Na^+^ and Cl^−^ is positively correlated with the degree of dehydration. The mortality rate of dogs with hypernatremia is 1.8 times that of the normal group (53, 54). In clinical application, I-FABP and CK-MB can increase within 0–6 h after the appearance of symptoms, which can be used for the early diagnosis of intestinal and cardiac involvement. At 48 h, CRP > 92.4 mg/L and cTnI > 0.5 ng/ml indicate that the survival rate is as low as 9%, which can be used for prognostic evaluation. The continuous monitoring of HCT and platelet count can guide the decisions of fluid therapy and blood transfusion, providing key references for timely adjusting the treatment plan.

**Table 1:** Hematological and inflammatory biomarkers in CPV infection.

Biomarker	Tissue origin	Changes in infection	Detection method	Clinical significance	Optimal testing time
Red blood cells (RBC)	Serum	Early elevation	Blood routine analysis	Indicate dehydration severity; guide fluid resuscitation therapy	0–24 h
Hematocrit (HCT)	Serum	Early elevation (hemoconcentration)	Blood routine analysis	Indicate dehydration severity; guide fluid resuscitation therapy	0–24 h
Hemoglobin (HGB)	Serum	Late decrease (aplastic anemia)	Blood routine analysis	Reflect chronic blood loss and bone marrow suppression	48–72 h
White blood cells (WBC)	Serum	Severe early leukopenia (<2,000 cells/mm^3^)	Blood routine analysis	Suggest immunosuppression; neutropenia correlates with poor prognosis	0–5 days
Platelet count	Serum	Decrease (<100,000/μl)	Blood routine analysis	Increase bleeding risk; associate with gastrointestinal mucosal injury	0–7 days
C-reactive protein (CRP)	Serum	>92.4 mg/L (sensitive for mortality prediction)	Serum biochemical assay	Acute-phase inflammatory marker; higher levels indicate more severe inflammation	0–48 h
Serum sodium (Na^+^)	Serum	Elevation (>150 mmol/L)	Serum electrolyte analysis	Correlate with dehydration; mortality in hypernatremic dogs is 1.8-fold higher	0–24 h
Intestinal fatty acid-binding protein (I-FABP)	Intestinal epithelial cells	5–10-fold elevation	Serum ELISA	Early marker of intestinal mucosal injury; predict mortality	Early infection (1–3 days)
Trefoil factor 3 (TFF-3)	Intestinal goblet cells	2–3-fold elevation	Serum ELISA	Reflect intestinal repair response; late prognostic indicator	Within 24 h of admission
Creatine kinase MB (CK-MB)	Myocardium	Elevation	Serum biochemical assay	Indicate acute myocardial injury	Immediate detection on admission
Cardiac troponin I (cTnI)	Myocardium	Sustained elevation	Serum immunoassay	Specific marker of myocardial cell necrosis; correlate with myocarditis severity	Immediate detection on admission
Salivary cholinesterase	Salivary glands	Moderate positive correlation with CRP	Salivary enzyme assay	Potential marker of disease severity (needs validation)	24–48 h after admission

## 3 Clinical diagnostic characteristics

Canine parvovirus infection shows stage-specific clinical manifestations. In the early infection stage (about 3–7 days), the main symptoms of sick dogs are hemorrhagic enteritis, including vomiting, diarrhea, anorexia, fever, and bloody feces. Complete blood count (CBC) shows a significant decrease in white blood cell count. If not treated promptly, the condition will deteriorate rapidly. In the middle stage of onset, the symptoms of vomiting and diarrhea in sick dogs are aggravated, the blood volume in excreta is increased, and the body’s resistance drops sharply (55). In the late stage of the disease, severe fluid loss leads to electrolyte imbalance, and even systemic acidosis, endangering life. In addition, some sick dogs may also develop non-suppurative myocarditis (56). The course of these cases is more dangerous, often lacking typical pre-stage symptoms, and the diagnosis is more difficult.

### 3.1 Rapid diagnostic techniques

The CPV colloidal gold test strip remains the most widely used rapid diagnostic tool. It offers advantages of simple operation, rapid detection, and high accuracy, making it the preferred choice in primary veterinary clinics. For operation, a small amount of canine feces is mixed thoroughly with normal saline, and 1–2 drops of the mixture are added to the sample well of the strip. Results can be interpreted within 5–10 min: a single color line indicates a negative a positive result, while two color lines signify a positive result. Notably, this method is influenced by factors such as fecal virus concentration and sampling timing. False-negative results may occur in early infection cases with low viral loads, necessitating comprehensive evaluation using supplementary detection methods (57, 58). The nano-gold lateral flow immunoassay (LFA-CPV antigen test) developed by Abousenna et al. offers a novel approach for detecting attenuated live CPV vaccines. Leveraging nano-gold labeling technology, this method enhances detection sensitivity and visualization, demonstrating significant value in vaccine quality control and immune efficacy assessment (116).

### 3.2 Common laboratory detection methods

#### 3.2.1 PCR detection technology

As the core of molecular diagnosis, PCR technology demonstrates extremely high sensitivity and specificity in CPV detection. With technological advancements, derivative techniques such as PCR diagnostic kits, nested PCR, and allele-specific PCR have been developed (59, 60). Among them, nested PCR can precisely distinguish CPV-2a/2b/2c subtypes. Sequencing of the VP2 gene at site 426 confirms the CPV-2c (D426E mutation). Real-time fluorescent quantitative PCR (qPCR) is 7,000-fold more sensitive than antigen detection, with Ct values inversely correlated with viral load. Studies show that vaccinated dogs have an average Ct value 4–6 units higher than naturally infected dogs, providing a quantitative basis for infection status (61). The SYBR Green-based qPCR method developed by Loor-Giler et al. enables rapid, sensitive, specific, and reproducible CPV quantification. Compared with traditional hydrolysis probe methods, it is more cost-effective and suitable for implementationin in primary laboratories, though further epidemiological studies and whole-genome sequencing are needed due to sample and sequencing limitations (62).

#### 3.2.2 Hemagglutination (HA) and Hemagglutination inhibition (HI) tests

HA and HI tests are classic serological methods for rapid diagnosis of CPV in fecal and tissue samples. The procedure involves isolating viral suspensions from fresh feces or intestinal contents of diseased or deceased dogs, followed by HA and HI assays (63). Although low-cost and simple, this method has relatively low sensitivity and is susceptible to non-specific interference, thus it is mainly used for preliminary screening (64).

#### 3.2.3 Virus isolation and identification

Virus isolation, the “gold standard” for confirming CPV infection (65), enables the isolate and identification of viruses from myocarditis and enteritis cases induced by CPV. However, it is time-consuming, requires high biosafety conditions, and has a success rate affected by sample preservation and disease stage-limiting its application in clinical rapid diagnosis and restricting it to research and difficult case confirmation.

#### 3.2.4 Enzyme-linked immunosorbent assay (ELISA)

ELISA technology has dual functions of detecting antibodies and antigens, featuring convenience, sensitivity, and high specificity. The double-antibody sandwich ELISA is a common method for detecting CPV antigens in canine feces, with good sensitivity and specificity, it is suitable for large-scale sample screening (66, 67).

#### 3.2.5 Other detection methods

Nucleic Acid Mismatch Enzyme Digestion (NMED): Developed by Weng et al., NMED has significant advantages in sensitivity, ease of operation, and equipment requirements over immunogold test strips and PCR, with lower costs. However, its complex DNA extraction process requires further optimization for practicality (68). LAMP-CRISPR/Cas12b Combined Technology: Chen et al. integrated loop-mediated isothermal amplification (LAMP) with CRISPR/Cas12b gene editing. This combined technology matches qPCR results but shortens detection time, enabling earlier identification of infected dogs. It offers an efficient solution for immediate CPV diagnosis and is expected to become a mainstream clinical technology (69).

With the continuous innovation of molecular diagnostic techniques, the diagnosis of CPV is advancing towards greater speed, sensitivity, precision, and intelligence. The application of these new technologies not only enhances the efficiency of disease diagnosis but also provides a scientific basis for formulating prevention and control strategies against CPV. In the future, with the combined application of multiple technologies and the development of artificial intelligence-assisted diagnosis, the diagnostic capabilities for CPV are expected to make significant advances.

## 4 Vaccination

Vaccination represents the most effective measure to prevent clinical CPV infection and canine-to-canine transmission. CPV vaccines are regarded as core vaccines by internationally influential professional associations such as the World Small Animal Veterinary Association (WSAVA) and the American Animal Hospital Association (AAHA). Irrespective of environmental conditions or geographical location, all dogs should be vaccinated to mitigate the global threat of this high-risk infectious disease. Canines acquire antibodies through two primary routes: transplacental transfer from the dam or ingestion of colostrum after birth. Colostrum serves as the main source of maternal CPV antibodies, with transplacental acquisition being minimal (70). Premature vaccination before 8 weeks of age may substantially weaken immune efficacy, as maternal antibodies can interfere with the vaccine, inhibit viral replication, and thereby reduce the capacity to generate protective immunity (71). Based on key considerations such as the core principles of vaccination and the impact of maternal antibodies, Table 2 summarizes the main recommendations for current canine parvovirus vaccination protocols, providing a standardized reference for clinical practice.

**Table 2:** The summary of types, characteristics and clinical recommendations of vaccines.

Vaccine Type	Preparation process	Immune effect	Advantage	Deficiency	Clinical recommended
Inactivated vaccine	Inactivated by physical and chemical methods such as high temperature, formaldehyde, β-propiolactone, with immune adjuvants.	Antibodies persist at low levels in vivo for 20 weeks, which can restrict the replication of the virus in the intestines and related lymphoid tissues.	High safety, uniform, simple manufacturing process, low cost.	The immunogenicity is reduced, requiring repeated vaccinations, and it is prone to immune failure due to interference from maternal antibodies.	recommended for pregnant bitches, rare canids, or dogs with immunocompromised functions.
Attenuated live vaccine	A live virus strain is obtained through attenuation, and it can replicate in the intestinal mucosa of animals after inoculation.	Induce the production of CPV serum neutralizing antibodies.	The immune effect is relatively significant, and it can stimulate a strong immune response.	After vaccination, the virus can be excreted in feces, which may lead to false positive results in detection; viral variation may reduce the efficacy of the vaccine.	Basic immunization: Start at 6–8 weeks of age, with a booster every 3–4 weeks until 16 weeks of age. High-risk dogs require annual booster immunizations.
DNA vaccine	A bacterial plasmid encoding a vaccine antigen, driven by a eukaryotic promoter, expresses the antigenic protein.	The replicon DNA vaccine encoding the VP2 gene can induce higher levels of virus-neutralizing antibodies and lymphocyte proliferation responses.	Antigen expression is more similar to the structure of natural proteins, and can induce cellular immunity.	Insufficient immunogenicity, requiring optimization through vector design and other methods.	Currently, it is only used in experimental research and is expected to be applied to the prevention and control of regional variant strains (e.g., Asian CPV-2a).
Subunit vaccine	Viral protein subunits (such as VP2 protein) or epitope peptides are prepared and can self-assemble into virus-like particles (VLPs).	Peptide vaccines can elicit a good humoral immune response.	It has high safety, is not interfered by maternal antibodies, and can be used as a marker vaccine to distinguish wild strain infections.	Epitope selection requires high conservation or coverage of multiple subtypes.	It is suitable for puppies with high levels of maternal antibodies or in regions where CPV-2c is prevalent.

### 4.1 Inactivated vaccines

Inactivated CPV vaccines are rarely available on the market because viral immunogenicity decreases during inactivation, requiring repeated administration in primary vaccination courses and annual booster shots. They are only recommended for exotic animals and pregnant bitches. Compared with modified live virus (MLV) vaccines, inactivated vaccines have a higher risk of immunization failure, likely because MLV vaccines possess stronger replicative capacity, enabling them to overcome residual maternal antibodies. However, inactivated vaccines offer higher safety than MLV vaccines, avoiding issues such as virulence reversion, and feature simple, uniform manufacturing processes with reduced production costs. The antibodies generated persist at low levels in the body for 20 weeks, providing protective effects against systemic infections by restricting viral replication to the intestine and intestinal-associated lymphoid tissues (72).

Traditional viral vaccines generally include inactivated vaccines and attenuated live vaccines. Inactivated vaccines are prepared by inactivating viruses through physicalchemical methods and emulsifying them with immune adjuvants, offering advantages such as good safety and simple manufacturing. The preparation process of inactivated vaccines is straightforward, using high temperature or inactivating agents (e.g., formaldehyde, β-propiolactone) to inactivate the virus. Inactivated CPV vaccines elicit immune responses in dogs, preventing intestinal viral replication, lymphopenia, leukopenia, clinical symptoms, and death (73–75). Sebbar et al. developed an inactivated adjuvanted CPV vaccine by culturing viruses, inactivating them, and adding adjuvants. Immunological experiments demonstrated high antibody titers, strong protective efficacy, and safety (75). However, inactivated vaccines have limitations such as slow antibody induction, inability to trigger cellular immunity, and the need for repeated administration to avoid unstable efficacy (76).

### 4.2 Attenuated live vaccines

Vaccination with attenuated live CPV vaccines remains an effective strategy for preventing CPV-induced enteritis. As early as 1982, live CPV vaccines were developed. Clinical trials showed that immunized dogs all developed CPV serum neutralizing antibody titers, although some exhibited local and systemic reactions (77). In fact, after vaccination, the live virus replicates in the intestinal mucosa of animals and is excreted in feces. Studies have shown that animals vaccinated with attenuated live vaccines can still shed CPV DNA in feces 28 days post-immunization, with potential transmission to exposed dogs (78, 79). Under such circumstances, traditional detection methods (ICAs, HA, or PCR) may yield false-positive results for CPV-2 or its nucleic acids in the feces of vaccinated dogs, leading to misdiagnosis (80).

Additionally, when live vaccines are administered in the presence of maternal antibodies, these antibodies can block active immunity in vaccinated puppies, causing immunization failure (81). Maternal antibodies become undetectable within 12 weeks of birth, so the initial vaccination of puppies should not be completed before 16 weeks of birth (82). A study demonstrated that oral immunization of puppies with attenuated live CPV-2b vaccine induced seroconversion even in the presence of maternal antibodies, without inducing stress in the animals (83).

CPV-2 is a rapidly evolving DNA virus with a mutation frequency of approximately 10^−4^ substitutions/site/year, approaching the evolution rate of RNA viruses (84, 85). The predominant circulating strain in China is CPV-2a, but the prevalence of CPV-2c is increasing, with new CPV-2a and new CPV-2b emerging successively (86, 87). The Pfizer Vanguard live-attenuated vaccine containing CPV-2 antigen, commonly used in China, has been shown to induce cross-neutralizing antibodies and provide protective immunity against virulent challenges from CPV-2a, CPV-2b, and CPV-2c (88). Although cross-protection has been confirmed, 48.42% of clinically positive CPV-2 samples were from vaccinated dogs, suggesting that CPV-2 vaccines used in northeastern China may fail to generate protective antibodies against heterogeneous CPV antigen types (89). In a study by Intervet, six beagles were vaccinated with Intervet’s attenuated CPV vaccine following the recommended protocol, while another six unvaccinated dogs served as controls. All dogs were then challenged with an oral dose of the CPV-2c strain. The results showed that the vaccinated dogs exhibited no clinical symptoms, and no virus was detected in their feces. In contrast, the control dogs developed typical symptoms of infection and shed the virus in their feces (90).

Field application of vaccines is influenced by multiple factors. For example, antigenic differences between CPV-2 and its variants may reduce the efficacy of CPV-2-based vaccines. It is recommended to use multivalent vaccines formulated with regionally prevalent CPV strains.

### 4.3 DNA vaccines

DNA vaccines, also known as nucleic acid vaccines, are based on bacterial plasmids encoding vaccine antigens, with expression driven by effective eukaryotic promoters. An effective DNA vaccine must enter the cytoplasm of cells at the injection site to induce *in vivo* antigen expression. The expressed antigens include necessary post-translational modifications, and are more similar to natural protein structures, enabling antigen presentation by major histocompatibility complexes (MHCs) and recognition by T cells (91). As early as 2005, the U.S. Department of Agriculture (USDA) approved the first DNA vaccine against West Nile virus for horses.

Insufficient immunogenicity remains the greatest challenge in the practical application of DNA vaccines. Strategies to enhance their immunogenicity include optimization of vector design, antigen codon optimization, the use of traditional and molecular adjuvants, electroporation (EP), and co-expression of molecular adjuvants. To increase antigen production and immunogenicity, a replicon-based DNA vaccine encoding the CPV VP2 gene has been developed, which drives heterologous antigen expression under the control of positive-strand RNA virus replicons. This vaccine elicits higher virus-neutralizing antibodies and lymphocyte proliferation responses than conventional CPV DNA vaccines and commercial CPV vaccines (92).

Considering rabies as a critical public health disease and the need for multivalent vaccines to protect dogs against both rabies and CPV (thereby avoiding multiple injections), a bicistronic DNA vaccine has been developed by subcloning the rabies glycoprotein and CPV VP2 genes into the bicistronic vector pIRES. Immunized dogs produced neutralizing antibodies against both rabies and CPV (93). Oral immunization delivers bacteria encoding DNA antigens to mucosal and systemic immune systems. A study successfully delivered a Sindbis virus replicon-based CPV DNA vaccine orally via *E. coli* DHSα, inducing sufficient CPV-specific humoral and cellular immune responses in immunized dogs (92). Although oral vaccines are cost-effective, easy to administer, and enable mucosal delivery, the lack of immune response due to gastrointestinal digestion remains the primary challenge to overcome.

### 4.4 Subunit vaccines

Since the first clinical trial of a melanoma antigen gene-1-derived peptide-based vaccine was reported in 1995, numerous clinical trials of peptide vaccines have been conducted (94). Epitope selection is a critical step in designing peptide-based vaccines, with the requirement for either highly conserved epitopes or multiple epitopes to cover various pathogen subtypes (95). Langeveld et al. fused B-cell and T-cell epitopes of CPV VP2 for recombinant expression. Animal immunization experiments showed that this peptide vaccine elicited robust humoral immune responses and could serve as a marker vaccine to distinguish wild-type virus infections during diagnosis (96). Dao et al. explored the potential of using a modified baculovirus expression system to produce CPV VP2 protein as a candidate antigen for CPV subunit vaccines. Based on the VP2 protein good immunogenicity, the study analyzed its application prospects in subunit vaccine development, highlighting advantages over traditional vaccines, such as higher safety and freedom from maternal antibody interference (97). In another study, the VP2 gene was subcloned into the pET-30a vector and co-expressed with the chaperone protein Tf16 in *E. coli* to achieve soluble expression of CPV VP2 protein. Under conditions of 250 mM NaCl and Ph 8.0, VP2 proteins self-assembled into virus-like particles (VLPs). Immunization of guinea pigs with 10, 30, or 50 μg of CPV VLPs showed that the 10 μg group exhibited a strong immune response, while the 50 μg group had maximum hemagglutination inhibition antibody and neutralizing antibody titers of 1:12288 and 1:6144, respectively (98).

### 4.5 Innovation of new vaccines driven by the dilemmas of traditional vaccines

Canine parvovirus (CPV) has maintained a high mutation rate since its emergence (61). Based on the molecular characteristics of its VP2 protein, it is classified into six genotypes: CPV-2, CPV-2a, CPV-2b, new CPV-2a, new CPV-2b, and CPV-2c. Currently, CPV-2c strains have a broader epidemic range (99–102) and exhibiting stronger pathogenicity than other subtypes. CPV outbreaks in adult dogs and vaccinated individuals occur frequently.

The D426E mutation in the VP2 capsid protein of the CPV-2c subtype represents the core mechanism underlying the reduced efficacy of traditional vaccines. This locus is situated within the viral epitope region, directly influencing the binding efficiency between the virus and the host TfR. The structural alteration not only enhances the virus’s affinity for canine intestinal epithelial cells but also induces conformational changes in the epitope, leading to a 4- to 8-fold decrease in the neutralizing antibody titers induced by traditional vaccines (5, 73, 103). The VP2 gene exhibits an annual mutation rate of 0.0045 substitutions/site, and this high evolutionary rate enabling significant genotypic variations in the virus every 2–3 years (104). Traditional vaccine development relies on pathogen isolation and culture, and the process from strain identification to vaccine production typically requiring 1–2 years, which is difficult to keep pace with the viral evolution rate. For instance, CPV-2c was first identified in 2000, but commercial vaccines containing CPV-2c antigens have remained limited, highlighting a notable lag in vaccine strain updating. Traditional multivalent vaccines (such as CPV-2 + 2b) do not fully consider regional strain differences in their design, leading to uneven protective efficacy. Epidemiological investigations have shown that in Europe and South America, where CPV-2c is predominant, the failure rate of traditional vaccines based on CPV-2/2b strains is as high as 34%, while that in Asian regions with CPV-2a prevalence is 12% (8, 99). Although Siedek et al. confirmed in 2011 that immunization of dogs with the commercial CPV-2 NL-35-D strain could confer resistance to CPV-2b and CPV-2c (105), a 2015 study by Beatriz et al. indicated that while CPV-2 and CPV-2b vaccines can alleviate clinical symptoms, reduce seropositivity rates, and decrease viral shedding, limitations such as small sample size, short follow-up time, and methodological issues currently render it impossible to determine whether they can provide effective protection against the CPV-2c subtype (106).

It is noteworthy that more than 80% of vaccine failures occur in puppies under 16 weeks of age, with 52% occuring before 10 weeks of age (107). Maternal antibodies (especially IgG) can bind to antigens of traditional vaccines, inhibiting the response of puppies’ own immune systems to vaccines. Studies have shown that when the maternal antibody titer is >1:64, the immunogenicity of traditional live-attenuated vaccines decreases by 40–60%, and the high mutability of CPV-2c further weakens the cross-protection in this case (71, 106, 108, 109). Traditional vaccines have limited efficacy against CPV-2c strains. To overcome this problem, a novel CPV-2c vaccine developed by Pearce et al. can circumvent high-level maternal antibodies in 4-week-old puppies, induce immune responses, and exhibit a strong correlation between antibody titer and protective efficacy. The immune mechanism may be related to the replication of vaccine virus at immune-privileged sites. It can rapidly induce immunity in puppies without maternal antibodies, and is safe for both dogs and cats, with no residual pathogenicity, genetic stability, and no reversion. The vaccine strain 630a is safe and effective for puppies older than 4 weeks of age, and subsequent vaccinations are still required as recommended (110).

In the evaluation of vaccine efficacy, cross-neutralization capacity is a key indicator. For CPV vaccines, since there are multiple variants of CPV, such as 2a, 2b, and 2c, whether the vaccine can produce cross-neutralization against different variants is directly related to its protective breadth and efficacy in practical application. The study on Vanguard C4 vaccine conducted by Raj et al. is of great significance. During the actual immunization process, when dogs are vaccinated with Vanguard C4 vaccine, the antigen components stimulate their immune system to produce CPV-specific antibodies. The study found that vaccinated dogs could produce effective immune responses against CPV 2a, 2b, and 2c variants isolated in Australia, with antibody titers reaching at least 1:160, far exceeding the protective titer (88). Even though the “Virus Neutralizing (VN)” antibody titer of CPV-2c variant was relatively the lowest compared to other variants, it still met the protective titer threshold. This indicates that when the vaccine stimulates the body to produce antibodies, the generated antibodies have broad recognition ability and which can exert neutralizing effects against different CPV variants, thus confirming its cross-protection capacity.

With the continuous emergence of multiple CPV variants, the protective efficacy of traditional vaccines against these variants is faces challenges. Therefore, novel CPV vaccines based on virus-like particles (VLPs)—which exhibit high immunogenicity and safety—and multi-epitope vaccines designed using computational methods and immunoinformatic to targeting highly conserved regions of the capsid protein (VP2) hold promise as candidates for CPV prevention (111–113). These new vaccines, tailored to prevalent CPV-2c strains, can better match viral characteristics and mitigate the impaired protective efficacy of traditional vaccines caused by viral mutations such as the D426E substitution in the VP2 capsid protein. This enables more effective protection for vulnerable populations like puppies during the infection window, reducing infection risk and disease severity.

Paul et al. designed a novel multi-epitope vaccine using immunoinformatic approaches. After screening for antigenicity and other criteria to identify conserved regions, they selected 10 non-allergenic, non-toxic, and highly antigenic epitopes. A 272-amino-acid vaccine candidate was constructed by linking these epitopes with GGS linkers, and incorporating the Salmonella flagellin FliC adjuvant and PADRE sequences. This vaccine, characterized by high antigenicity and stability, can be expressed in *E. coli* K12 and shows no similarity to canine proteins, holding potential for preventing canine parvoviral enteritis. However, its efficacy requires further experimental validation (114). In a 2023 study, Zhao et al. constructed and evaluated recombinant CPV VLPs displaying major antigenic epitopes of giant panda-derived canine distemper virus (CDV). Mouse immunization experiments demonstrated that these VLPs induced high levels of CPV- and CDV-specific antibodies as well as neutralizing antibodies, while also activating splenic lymphocyte proliferation. The results indicated that these chimeric VLPs could serve as bivalent vaccine candidates against CPV and CDV in giant pandas (115).

At present, most commercial vaccines use CPV-2 or CPV-2b strains as antigenic strains, which have significant antigenic differences from the widely prevalent CPV-2c strains. This discrepancy has led to the failure to effectively contain the transmission of canine parvovirus disease. In view of this, the superposition of factors such as the high mutation rate of CPV, differences in regional epidemic strains, and maternal antibody interference has further exacerbated the complexity of disease prevention and control, and has also highlighted the urgency of accelerating the development of novel vaccines and optimizing immunization strategies, which is crucial for improving prevention and control efficiency.

## 5 Conclusion

CPV is a significant pathogen in veterinary medicine, posing a severe threat to the health of dogs. Its continuous evolution, particularly specific mutations in the VP2 capsid protein, has led to the emergence of multiple antigenic variants, such as CPV-2c. CPV-2c has become the dominant strain in some regions, thereby altering the epidemiological characteristics and pathogenicity of the disease.

Biomarkers play a crucial role in the diagnosis, treatment, and prognostic assessment of dogs affected by CPV. Changes in blood components, including red blood cells, white blood cells, platelets, and serum biochemical indicators, as well as elevated CRP levels, can reflect different stages of the disease, the degree of inflammation, and the severity of the condition. For instance, dynamic changes in red blood cell parameters can help veterinarians understand dehydration and anemia in affected dogs during the disease process. Intestinal biomarkers, such as I-FABP and TFF-3, are indicators of intestinal injury and repair, while cardiac biomarkers, including CK-MB, cTnI, and H-FABP, are useful for assessing myocardial damage caused by CPV. Although salivary cholinesterase alterations show potential as a severity assessment biomarker, its reliability requires validation through larger-scale studies, given the limited sample sizes in current research limited sample sizes in current research.

In vaccine research, although some breakthroughs have been achieved, challenges remain. Novel CPV-2c vaccines are capable of overcoming the interference of high levels of maternal antibodies in 4-week-old puppies and inducing immune responses. Reverse vaccinology has provided an effective approach for designing multi-epitope vaccines. However, factors such as antigenic drift of CPV and the immaturity of the immune system in puppies still limit vaccine efficacy. For example, antigenic differences between CPV-2c and traditional vaccine strains can lead to a significant reduction in neutralizing antibody titers induced by traditional vaccines, contributing to a relatively high vaccination failure rate in puppies under 16 weeks of age.

In summary, continuous efforts are needed in multiple aspects to effectively control CPV-related diseases. Future research should focus on closely monitoring viral mutations to better understand the characteristics of new variants; further exploring biomarkers to improve the accuracy of early diagnosis; developing more effective, safer, and strain-specific matched vaccines; and innovating diagnostic methods to achieve precise identification of different infection states. These efforts will help to mitigate the harm caused by CPV to dogs and promote the healthy development of the dog breeding industry.
