# Correction: Decoding canine parvovirus: biomarkers for diagnosis and advances in vaccine development to address emerging challenges

**DOI:** 10.3389/fvets.2025.1684220

**Published:** 2025-09-01

**Authors:** Hao Li, Shengnan Li, Ye Pan, Qiumei Shi

**Affiliations:** ^1^The Laboratory Animal Research Center, Jiangsu University, Zhenjiang, China; ^2^School of Medicine, Southeast University, Nanjing, China; ^3^Hebei Provincial Key Laboratory of Preventive Veterinary Medicine, College of Animal Science and Technology, Hebei Normal University of Science and Technology, Qinghuangdao, China

**Keywords:** canine parvovirus, biomarker, enteritis, myocarditis, vaccine

Affiliation 1, The Laboratory Animal Research Center, Jiangsu University, Zhenjiang, China, was erroneously assigned to Qiumei Shi.

This affiliation has now been removed for author Qiumei Shi.

The original version of this article has been updated.

